# Lessons learned: Commentary on patient harm related to COVID-19 prevention policies at a rural academic tertiary care facility

**DOI:** 10.1016/j.amsu.2020.06.036

**Published:** 2020-07-10

**Authors:** Abhinav Mittal, Kyle Davis Chapman, Michael Forte, Hatim Al-Jaroushi

**Affiliations:** West Virginia University Department of Medicine, Section of Pulmonary, Critical Care & Sleep Medicine, United States

**Keywords:** COVID-19, Healthcare policy, Adverse events, Patient safety, Rural medicine

## 1 Introduction

While the toll COVID-19 exacts on the global population will be measured in confirmed cases and mortalities, its legacy will be decided by our response. Each healthcare system must sort the frenzy of fragmented new published data in real time to create coherent policies specific to their practice. They must maintain standards without compromising safety of employees or patients. With uncertainties of resource scarcity, we must prepare to possibly address ethical incongruencies in the principals of autonomy, beneficence, justice, and non-maleficence applied to the individual patient and the community as a whole.

We evaluate the inadvertent impact of COVID-19 prevention policies made at a rural academic tertiary care center and their associated unintended adverse events.

## 2 No harm event

Policy 1: Anyone with COVID-19 contact/symptoms must self-quarantine for 14 days or return with negative testing.

*Description*: Within weeks of COVID-19 emergence in the US, 2/8 Pulmonary and Critical Care (PCCM) faculty were forced into quarantine. Their initial Polymerase Chain Reaction (PCR) samples were lost – keeping these highly valuable individuals from serving. In fact, they returned only once rapid PCR testing became available within our own institution.

*COVID-19 Impact*: PCCM specialists are a rare commodity. Though the concern of an infected provider spreading disease to critically ill patients cannot be overstated, the loss of a quarter of our workforce exacerbates the burden of a pandemic on an already stressed system.

*Outcome:* Fortunately, these quarantined PCCM faculty kept busy. They spearheaded the task of expanding telemedicine capabilities and transitioned all of our outpatient PCCM clinics into virtual visits. Telemedicine allowed us to remotely provide specialty care to the patients most prone to harm. This may potentially save far more lives than they could in person.

### 2.1 Sentinel events

Policy 2: COVID-19 confirmed or Person Under Investigation (PUI) must be admitted to airborne isolation.
Policy 3: Non-essential diagnostics/therapeutic interventions should be delayed. When essential, airborne isolation should be used.

*Description*: We had two instances of delayed cardiac catheterizations for acute coronary syndrome (ACS). While both patients eventually received cardiac catheterization, a lack of specificity in the catheterization policy manifest in confusion. Luckily, both had no lasting harm.

Unfortunately, not everyone was that lucky. One patient was admitted to a sister hospital for Non-ST Elevation Myocardial Infarction as a PUI and started on systemic anticoagulation. She developed new onset bilateral lower extremity weakness but was found to have an epidural hematoma from C6 – T7 two days later. The patient was transferred to us where our neurosurgeons operated in airborne isolation.

*COVID-19 Impact*: We generate and refine a differential diagnosis by patient *history, physical assessment, and diagnostic testing*. Given uncertainties of transmission, risk of aerosolization, and shortage of Personal Protective Equipment (PPE), suddenly clinicians became limited in their ability to obtain these. Because of asymptomatic carriers and prevalent community spread, many admitted patients became PUIs.

*Outcomes:* As a rural institution with relative geographic isolation, we had the benefit of lead time. Though we enhanced our PPE and airborne capable rooms, we did not anticipate everything. A time sensitive medical emergency like ACS had to suffer brief but not-insignificant delays due to conflict between departmental and institutional policies with no clear line of command to resolve. For the patient who developed epidural hematoma, limited neurologic assessments likely related to her PUI status delayed detection, imaging, and timely intervention of a well-known risk of anticoagulation. This woman will likely be ventilator dependent lifelong.

In response, we have now created a clear protocol on ACS care in COVID-19 with discrete inclusion/exclusion criteria. We also have created a centralized COVID-19 Command Center that is staffed continuously to resolve issues.

## 3 Never event

Policy 4: COVID-19 confirmed or PUI patient with respiratory failure should be intubated without trial of High Flow Nasal Cannula (HFNC) or Non-Invasive Positive Pressure Ventilation (NIPPV) due to risk of aerosolization.
Policy 5: COVID intubations will be done with passive pre-oxygenation via Rapid Sequence Intubation (RSI) without Bag Mask Ventilation (BVM).

*Description:* An elderly PUI with history of congestive heart failure (CHF) and end stage renal disease presented with hypotension and hypoxia during outpatient hemodialysis. She developed worsening respiratory failure and decision was made to intubate. While awaiting onset of RSI medications under apnea, she became rapidly hypoxic and suffered cardiac arrest. Though return of spontaneous circulation was achieved, she did not survive the night.

*COVID-19 Impact*: Clinical decision making is guided by evidence based practices whenever possible. A novel virus with no “standard of care” coupled with ubiquitous fears can create an environment of chaos. How does one balance the axiom of beneficence with the principals of non-maleficence to both patients and providers, autonomy of healthcare professionals, and justice to society?

*Outcomes:* This patient encounter was an unmitigated tragedy for the patient, her bereaved, and the treatment team. A bad milieu of co-morbidities diminished her ability to withstand illness. Despite evidence supporting use of NIPPV in CHF, risk for SARS-CoV-2 aerosolization limited its use. With no evidence based practices to guide safe intubation, policy based on expert opinion left little room to address the “shades of grey.”

Given this experience, we revised policy to allow HFNC, NIPPV, and BVM per provider discretion. We have a new standardized manner of intubation using COVID-19 kits. We also developed a curriculum and rapidly trained all clinical personnel in COVID-19 CPR and intubation. The collective efforts of a multidisciplinary group implemented these measures within 2 days of this case.

## 4 Conclusion

Every individual discussed suffered a COVID-19 related set-back, but was ultimately PCR negative. Though test sensitivity is imperfect, suspicion was also low. Global social distancing speaks to humanity's ability to unite. As more is learned, we continue to modify our approach to best serve our patients. All the above policies have been amended and inspired more innovation. Ultimately, we believe that clinical decisions should be made as per standard of care irrespective of COVID-19 status unless there is a clear reason to deviate. The best way to uphold the ethical principles is to think critically before deviating from evidence based medicine.

## Ethical approval

Nothing to declare.

## Sources of funding

Nothing to declare.

## Author contribution

Abhinav Mittal: Commentary concept, data collection, data analysis, writing the original draft, reviewing and approving final draft.

Kyle Chapman: Commentary concept, data collection, data analysis, reviewing and approving final draft.

Mike Forte: Commentary concept, data collection, data analysis, reviewing and approving final draft.

Hatim Al-Jaroushi: Commentary concept, data collection, data analysis, reviewing and approving final draft.

## Registration of research studies

Nothing to declare.

## Guarantor

All authors of this commentary: Abhinav Mittal, Kyle Chapman, Mike Forte, Hatim Al-Jaroushi.

## Consent

No patients are discussed in any detail and no health protected information is discussed because only general statements are made that are in no way able to be connected to any specific patient. This is simply a commentary that does not detail anything specific.

## Provenance and peer review

Not commissioned, externally peer reviewed.

## Declaration of competing interest

Nothing to declare.

